# Consistent defined threshold and equity in health

**DOI:** 10.1186/s40199-015-0097-6

**Published:** 2015-02-10

**Authors:** Shekoufeh Nikfar, Zaheer-Ud-Din Babar, Rassoul Dinarvand, Abbas Kebriaeezadeh, Mohammad Abdollahi

**Affiliations:** Department of Pharmacoeconomics and Pharmaceutical Administration, Faculty of Pharmacy, Tehran University of Medical Sciences, Tehran, Iran; School of Pharmacy, Faculty of Medical and Health Sciences, University of Auckland, Auckland, New Zealand; Faculty of Pharmacy, and Pharmaceutical Sciences Research Center, Tehran University of Medical Sciences, Tehran, Iran

In measuring health, economic evaluations, the threshold is an important concept. It signifies the value of health gain and a new intervention is considered satisfactory if its price falls below a certain threshold [1].

Results are usually reported in an incremental cost-effectiveness ratio (ICER). The ICER stands for the additional costs per additional health unit produced by one intervention in comparison to another. A common tool for measurement is the quality-adjusted life-year (QALY). QALY encompasses both length and quality of life, which is based on utility. Likewise, various organizations and governmental bodies such as the National Institute for Health and Clinical Excellence (NICE) in the UK, Swedish Pricing and Reimbursement Board, the Pharmaceutical Benefits Advisory Committee in Australia, Dutch Health Care Insurance Board (CVZ) in The Netherlands have adopted certain threshold values. This adoption of threshold optimizes the process of allocation of health care resources [2].

Reimbursement decisions and allocation of health care resources is evolving in developing nations. Though cost-effectiveness analysis is increasingly being used for reimbursement mechanisms in developing countries, however, there is no consistent defined threshold. Most developing countries have adopted one to three times of their local gross domestic product (GDP) per capita as a threshold. This is also recommended by the World Health Organization (WHO). However, it is argued that the range of 1 to 3 folds is a wide range and hence it is imperative to evaluate the exact QALY threshold especially with regards to public preferences [3].

Another tool for estimating threshold is to conduct willingness to pay (WTP) studies. However, WTP for a QALY is inconsistent and dependent on the size, duration, and type of the health gain [4]. Therefore, it is considered that WTP is directly correlated with the nature and burden of disease. For example, WTP would increase if the patient suffers more of a certain disease.

Nowadays, most of the new medicines to treat cancer, asthma, arthritis rheumatoid, central nervous system diseases and inflammatory bowel disease are more expensive. Economic burden of these diseases is very high because of direct medical costs as well as disabilities resulting in indirect cost. Cost of illness increases especially when patients have to make out of pocket payment for medicines [5-9]. This is compounded by the fact that newer biotechnology medicines are very expensive and even their generic versions are not affordable [10,11].

In this context, consistent threshold would pose a problem, equity will be affected and as for most of expensive drugs they will be rejected from the reimbursable list of medicines [12,13]. This would defy the access as newer medicines are necessary for patients with more complicated illnesses [14]. The role of government is to implement a policy for equal opportunities that is healthy aligning with the equity in health [15,16].

Considering this approach, WTP may be a better solution for decision making while calculating a threshold. WTP rationally expresses the health state of patients while taking into consideration patients’ views about the health. Though the calculation of WTP is time consuming and sometime it’s feasibility questioned too.

Evaluation of strengths and limitations of differing estimations of thresholds is vital. This helps to find appropriate monetary values for QALY. More pragmatic researches are needed in this area and work toward a higher level of reliability in decision-making is required.

Efficiency and allocations in healthcare are emerging concerns in the field of pharmacoeconomics and pharmaceutical policy. This cross-journal series will disseminate new ideas, methods, and findings of applied pharmacoeconomics in implementation of pharmaceutical policies.
